# New insights into the immunologic role of oligodendrocyte lineage cells in demyelination diseases

**DOI:** 10.7555/JBR.36.20220016

**Published:** 2022-04-28

**Authors:** Hui Li, Yang Chen, Jianqin Niu, Chenju Yi

**Affiliations:** 1 Research Centre, the Seventh Affiliated Hospital of Sun Yat-sen University, Shenzhen 518107, China; 2 Department of Histology and Embryology, Chongqing Key Laboratory of Neurobiology, Brain and Intelligence Research Key Laboratory of Chongqing Education Commission, Third Military Medical University, Chongqing 400038, China

**Keywords:** oligodendrocyte, oligodendrocyte precursor cell, demyelination disease, multiple sclerosis, immunology

## Abstract

Oligodendrocyte lineage cells (OL-lineage cells) are a cell population that are crucial for mammalian central nervous system (CNS) myelination. OL-lineage cells go through developmental stages, initially differentiating into oligodendrocyte precursor cells (OPCs), before becoming immature oligodendrocytes, then mature oligodendrocytes (OLs). While the main function of cell lineage is in myelin formation, and increasing number of studies have turned to explore the immunological characteristics of these cells. Initially, these studies focused on discovering how OPCs and OLs are affected by the immune system, and then, how these immunological changes influence the myelination process. However, recent studies have uncovered another feature of OL-lineage cells in our immune systems. It would appear that OL-lineage cells also express immunological factors such as cytokines and chemokines in response to immune activation, and the expression of these factors changes under various pathologic conditions. Evidence suggests that OL-lineage cells actually modulate immune functions. Indeed, OL-lineage cells appear to play both "victim" and "agent" in the CNS which raises a number of questions. Here, we summarize immunologic changes in OL-lineage cells and their effects, as well as consider OL-lineage cell changes which influence immune cells under pathological conditions. We also describe some of the underlying mechanisms of these changes and their effects. Finally, we describe several studies which use OL-lineage cells as immunotherapeutic targets for demyelination diseases.

## Introduction

Oligodendrocyte precursor cells (OPCs) are the fourth glial population in the central nervous system (CNS)^[1–3]^. While it is widely accepted that OPCs primarily mature into oligodendrocytes (OLs) during CNS development as well as into adulthood, there are various distinctions between OPCs and OLs. OPCs have dual-purposes, such as morphology during migration and have multiple processes after the cessation of migration. By contrast, OLs appear to predominantly extend multiple processes to ensheath axons and form myelin segments. However, OPCs express platelet-derived growth factor receptor alpha^[4–5]^, chondroitin sulfate proteoglycan 4^[6]^, the cell surface ganglioside epitope A2B5^[7]^, G protein-coupled receptor 17^[8]^, and connexin47^[9]^ during their early development; whereas, OLs express choline-specific glycerophosphodiester-phosphodiesterase^[10]^, breast carcinoma amplified sequence 1^[11]^, 2',3'-cyclic-nucleotide 3'-phosphodiesterase (CNPase)^[12]^, adenomatous polyposis coli^[13]^, surface antigen O1^[14]^, myelin basic protein^[14]^, myelin oligodendrocyte glycoprotein (MOG)^[15]^, and myelin-associated glycoprotein^[16–17]^ during maturation.

Perhaps more importantly, there are functional differences between OPCs and OLs. OLs have traditionally been regarded as a cell population that plays a major role in the myelination of the CNS. OLs therefore support axon integrity by secreting brain-derived neurotrophic factor (BDNF), nerve growth factor, glial cell-derived neurotrophic factor, and insulin-like growth factor to promote neuronal development, while expressing the monocarboxylate transporter 1 to transfer lactic acid into axons^[18]^. OLs also maintain axonal ions and morphological homeostasis by expressing the K^+^ channel Kir4.1^[19]^ and by regulating CNPase levels. Throughout CNS development, OPCs differentiate into OLs to form myelin. In adult multiple sclerosis (MS) which can affect the brain and spinal cord, OPCs can be activated to proliferate and migrate into areas with lesions where they play an important role in remyelination^[20]^. Incidentally, OPCs appear to also have a role in glia scar formation^[21]^ and in blood-brain barrier regulation^[22]^. Studies have also found that OPCs can differentiate into astrocytes and neurons when faced with acute injury, indicating that OPCs have lineage plasticity^[23]^.

Another important function that has stimulated interest in OPCs is the immunological role of OL-lineage cells. Over the past few decades, an increasing amount of research has focused on the immunological characteristics of OL-lineage cells. Initially, OL-lineage cells have been found to respond to immune changes and therefore can be a target of immune attack^[24]^. However, many studies have also found that these cells can respond to immune changes by transforming themselves to regulate our immunity responses^[25]^. These transformations appear to depend on the specific stage and lineage which leaves us with a number of unknowns. Here, we review how OL-lineage cells can act as both "victim" or "agent" in the CNS, and how OL-lineage cells undergo different changes and effects at various stages, as well as describe the mechanisms which underlie these changes and effects.

## Immune changes in OL-lineage cells in disease and the effects

OL-lineage cells appear to play "victim" when influenced by immune changes in the CNS under pathological conditions like demyelinating diseases. Different immune cells have different impacts on OL-lineage cells. For example, T lymphocytes, B lymphocytes, and myeloid cells regulate OPCs predominantly by secreting cytokines, chemokines, and other factors. Proinflammatory T cells (T helpler 1 cell/T helpler 17 cells, Th1/Th17) and M1 myeloid cells supernatants have a direct cytotoxic effect on OPCs which are described as human A2B5^+^ neural progenitors, and lower the number of O4^+^ and GalC^+^ OLs. In cuprizone (CPZ)-induced demyelination, an experimental model used to mimic the demyelinating features of diseases, MOG specific CD4^+^ T cells polarize into Th17 cells and migrate into the corpus callosum, which impairs remyelination^[26]^. However, in MS, which is the most common demyelinating diseases involving OL-lineage cells, OPCs numbers increase during the acute stage but decrease at a chronic stage^[27]^. The number of OPCs expressing caspase-3/7 increases which induce cell death; however, the increase of both interleukin-11^[28]^ and interleukin-17A (IL-17A)^[29]^ actually enhances survival and improves OPCs differentiation, thereby actually promoting remyelination.

Following anti-CD3/CD28 treatment, CD4^+^ and CD8^+^ T cells are activated and secrete vesicular endothelia growth factor α (VEGFα) which encourages OPCs to proliferate by promoting cell cycle transition from the G1 phase toward the S phase^[30]^. OPCs apoptosis is mediated by M1 myeloid cells depending upon tumor necrosis factor-α (TNF-α), rather than proinflammatory T cells. Th1 and M1 myeloid cells can however also impair OPCs differentiation by regulating cytokine secretion and growth factors from astrocytes. However, Th2 and M2 macrophages have no significant impact on OPCs differentiation^[31]^. Similarly, M1 microglia mediate inflammatory injury, whereas M2 microglia produce an antithetical effect^[32]^. T cells produce IL-17 or interferon-gamma (IFN-γ) which disrupt remyelination independent of OPCs differentiation status^[33]^. Likewise, B lymphocytes, when derived from MS patients, can induce OLs death, depending on whether these B cells are unstimulated or stimulated by CD40L. However, this effect may also be dependent upon changes in microglia or astroglia activity and may not be mediated by immunoglobulins^[34]^.

**Figure 1:**
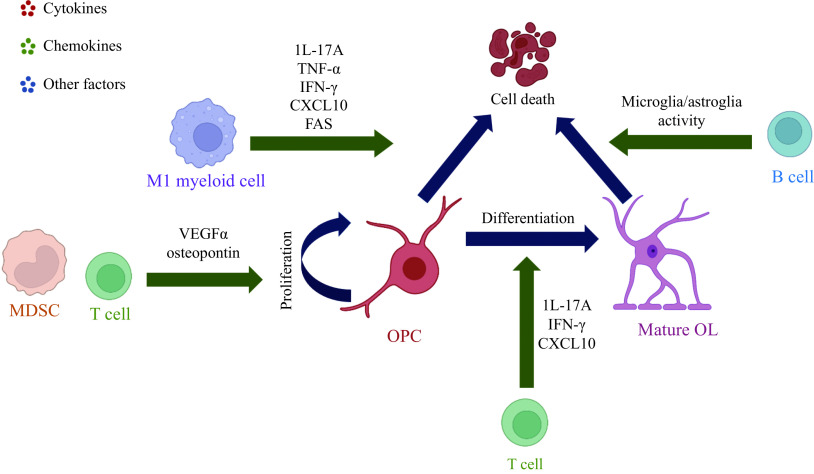
Schematic of immune system mediating proliferation, differentiation, and cell death of OL-lineage cells.

For example, C-X-C motif chemokine ligand 10 (CXCL10) signaling to C-X-C motif chemokine receptor 3 in response to IFN-γ promotes OPCs apoptosis through a caspase-dependent mechanism^[35]^. MS specific CD49d^+^CD154^+^ lymphocytes also interact with OL-lineage cells to inhibit mature OLs myelination by increasing proinflammatory chemokines/cytokines secretion and promote microRNA-665 synthesis by downregulating polymeraseⅡ which impedes remyelination^[36–37]^. Myeloid-derived suppressor cells secrete osteopontin to protect OPCs against immune attack and promote their proliferation and mobility toward the MS lesion and mature into OLs^[38]^. When exposed to nutrient deficiencies such as low glucose levels, OLs show increased survival rate^[39–40]^. This is in contrast to BDNF deficiency, which restricts OPCs proliferation following demyelination^[40]^. Together, current studies have uncovered various immune changes by OL-lineage cells during disease progression and have characterized their effect, which suggests that OL-lineage cells can play a "victim" role in the CNS immune system (***Fig. 1***).

## Immunological changes in OL-lineage cells under pathological conditions

As well as being affected by immune cell changes, there are immunological changes which OL-lineage cells go through under various pathological conditions. For example, in CPZ-induced demyelination, OPCs are stimulated to express cytokine IL-1β and C-C motif chemokine ligand 2 (CCL2)^[41]^. IL-1β is a pro-inflammatory cytokine involved in lymphocytes recruitment as part of our innate immunity and in Th1/Th17 cells which are associated with adaptive immunity^[42]^. CCL2 is best known for regulating macrophage recruitment and in polarization^[43–44]^. IFN-γ released from T cells under immunological pathological conditions induces upregulating of genes encoding antigen processing and presents molecules, *e.g.*, major histocompatibility complex class Ⅰ (MHC Ⅰ), in OPCs. This leads to OPCs engulfment, processing and presentation of antigens, and activation of CD8^+^ cytotoxic cells, which in turn affects OPCs survival^[33]^. Additionally, the TNF family induces OPCs to express CD273 and CD274 which can act like antigen processing molecules *in vitro*. This therefore activates T lymphocytes to produce other cytokines and attack OPCs^[45]^. The release of inflammatory cytokines from OPCs are reduced following IL-17 deletion in autoimmune demyelination^[46]^.

In line with the immunological changes presented by OPCs, OLs also express immune factors which can actually change their own immunological state. Cytokine IL-18 is expressed by OLs during MS^[47]^, which indicates that OLs have the capacity to induce IFN-γ or Th2 associated cytokines. Studies have also found a series of inflammasome sensor molecules including NLR family pyrin domain containing 1 (NALP1) and NALP3 expressed by OLs in normal-appearing white matter in MS^[48]^. OLs are found to express CCL2, a chemokine known to regulate microglia and monocyte migration, following *in vitro* treatment of IFN-γ^[41,49]^. Besides, CXCL10, CCL3, and CCL5 are also expressed in OLs upon IFN-γ stimulation. These chemokines were known to induce chemotaxis by inducing recruitment and migration of immune cells^[50–52]^. While OPCs express MHC Ⅰ in response to IFN-γ stimulation, cultured OLs from rat nerve express MHC Ⅱ upon stimulation by dexamethasone^[53]^. Semaphorin 3A (SEMA3A) and 3F (SEMA3F) are upregulated by OLs within the active MS lesion^[54]^, and SEMA3A has been shown to induce microglia apoptosis^[55]^. Together, these findings demonstrate that OL-lineage cells express immune factors and suggest that they possess some immunomodulatory capacity.

## OL-lineage cells regulate immunity under pathological conditions

OL-lineage cells can also act as "agent" in our immune responses to neuroinflammatory diseases. This means that OL-lineage cells are able to regulate immunity *via* different pathways although, these predominantly including interactions with microglia^[56]^ and T cells^[57]^. In MS, OPCs release exosomes^[58–59]^ which contain microRNAs such as miRNA-145. These are highly expressed components in OPCs and are targeted at microglia. Microglia internalize the exosomes through macrocytosis and are consequently activated^[58]^. Simultaneously, OLs are stimulated to release IL-18^[47]^ which induces adaptive immune cells^[60]^ as well as microglia^[61]^, to produce IFN-γ. As has been mentioned, the IFN-γ released may then contribute to oligodendrocyte damage^[33]^. Furthermore, OLs express membrane glycoprotein CD200 which binds the CD200 receptor on microglia and exerts an inhibitory effect on pro-inflammatory microglia activation *via* CD200-CD200R interaction^[62]^.

Additionally, OL-lineage cells have the capacity to regulate the immune system through interactions with T cells. For example, pathological OPCs in MS are stimulated to express MHC Ⅰ and are involved in antigen cross-presenting which activates CD8^+^ T cells. As well as expressing MHC Ⅰ, antigens presenting CD273 molecules and CD274 expressed in OPCs also regulate T lymphocytes by promoting (or inhibiting) proliferation^[45,63]^. However, OLs in MS can express IL-18, which co-stimulates Th1 or Th2 in the presence or absence of IL-12^[64]^. When IL-12 is present, IL-18 induces T cells, B cells, and NK cells to produce IFN-γ and co-stimulates Th1 cells. Instead, IL-18 induces Th2-related cytokines from T and NK cells which then initiate an anti-inflammatory effect in the CNS^[64–66]^. OLs can also function as antigen presenting cells because of their MHC Ⅱ expression which can be induced by the demyelination inducer dexamethasone. It is widely held that MHC Ⅱ is involved in presenting an antigen that activates CD4^+^ T cells^[53]^.

## Oligodendroglia-mediated immunological effects on other cellular pathologies

Since OL-lineage cells are considered immunomodulators in the CNS, the immunological effect which is mediated by these populations is thought to be substantial. OPCs secrete exosomes or bind surface proteins to initiate immune responses. In demyelinated lesion areas, NG2^+^ OPCs, Oli-neu cells (which is an oligodendroglia cell line), and primary OL-lineage cells secrete exosomes that are internalized by microglia. This action therefore enhances phagocytic ability of microglia^[58–59,67]^. OPCs then cross-present ovalbumin and induce CD8^+^ T cells to secrete TNF-α which in turn affect OPCs survival. The cross-presenting antigen of OPCs also includes perforin and granzyme B which can both induce caspase cascades in the target cells and eventually leads to cell death^[33]^.

**Figure 2:**
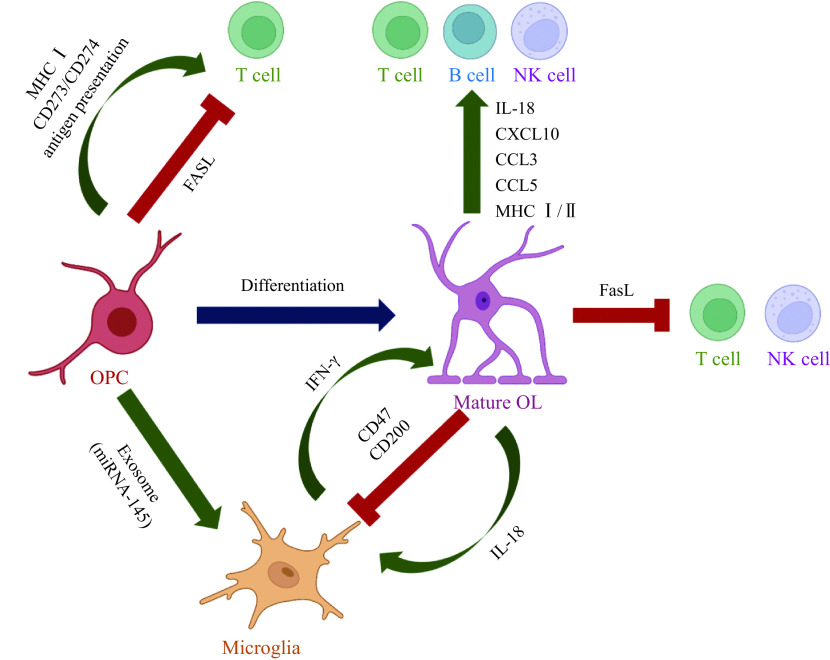
Schematic of oligodendroglia lineage cells regulating the immune system.

OLs can also regulate the immune system by secreting various factors. In MS, OLs are stimulated to release cytokine IL-18 which increases the production of IFN-γ from microglia, thereby promoting M1 microglia polarization^[68]^ and simultaneously regulating the number of OPCs^[33]^. Chemokines CXCL10, CCL3, and CCL5 are released from IFN-γ stimulated OLs^[49]^ whose primarily function is to induce the recruitment of leukocytes and the migration of T cells, NK cells, and dendritic cells^[52]^. Furthermore, OLs regulate the immune system with surface binding proteins. OLs express the inflammasome component  NLR family CARD domain containing 4, which is essential for T lymphocytes and NK cells to release IFN-γ^[69]^, one of the main immune components which stimulates macrophages to express inducible nitric oxide synthase^[68]^. Tetraspanin CD81^[70]^ and CD82^[71]^ expressed by OLs induce MHC Ⅰ clustering^[72]^, but when accompanied with CD9 this can also induce MHC Ⅱ clustering^[73]^. These MHC clusters differentially modulate T and B cells. CD47 expressed in OLs plays a suppressive role on microglia and macrophages through interactions between CD47 on myelin and signal regulatory protein-α (SIRPα) on phagocytes^[74]^. Additionally, OLs express Fas ligand^[75]^ which triggers the activation of Fas expressed T and NK cells, thus constraining inflammation^[76]^. Collectively, OL-lineage cells regulate immune cells derived from peripheral blood and those which reside in the CNS by secreting various factors, binding surface proteins, and delivering exosomes (***Fig. 2***).

## Mechanisms underlying immunologic changes in OL-lineage cells

The mechanisms underlying the aforementioned changes and effects in OL-lineage cells have been elucidated in recent years. For instance, activated T cells promote OPCs proliferation by releasing VEGFα. Released VEGFα functions by binding the specific receptor VEGFR2 which is expressed in OPCs^[30]^. Studies have identified a specific bioactive hyaluronan fragment (bHAf) that can block OPCs maturation and OL-lineage cells myelination with an immune-tolerance-like approach. These bHAfs activate protein kinase B (PKB, also known as AKT) *via* the Toll-like receptor 4/TIR domain-containing adapter-inducing IFN-β pathway, which subsequently activates the transcription factor, Forkhead Box O3 (FoxO3) which constrains the maturation of OL-lineage cells at the OPCs stage^[77]^. This suggests that the TLR/AKT/FoxO3 pathway is implicated in OPCs' capacity to remyelinate. Furthermore, TLR3 is involved in the upregulation of CCL2 and CXCL10 in OLs in response to IL-1β^[78]^. OPCs have also been found to increase their expression of caspase-3/7, indicating that the activation of CD8^+^ cytotoxic T cells affects cell survival *via* the contact-dependent Fas-FasL interaction pathway^[33]^.

Besides, OPCs have been found to function as antigen presentation cells which activate CD8^+^ cytotoxic cells* via* cross-presentation. Through the stimulation of IFN-γ, OPCs express MHC Ⅰ which helps to engulfment, process and present ovalbumin which eventually results in CD8^+^ T cell activation. OPCs' ability to cross-present antigens can be inhibited by the downregulation of LRP-1 expression on OPCs. When LRP is inhibited by its inhibitor RAP, the expression of MHC Ⅰ in OPCs substantially decreases and the activation of CD8^+^ T cells consequently decreases^[79]^. However, the mechanisms by which OL-lineage cells function as a modulator have not been studied thoroughly.

Under the experimental autoimmune encephalomyelitis (EAE) model, OPCs have been induced to express inflammatory genes in response to IL-17, which eventually leads to cell death. One study also has found that the reduction of NF-κb activator1 (Act1) in OL-lineage cells, which is a key transducer of IL-17 receptors (IL-17R) signaling, attenuates EAE pathology^[80]^. A further study explored an IL-17R recruitment of Fas-activated death domain through the SEF/IL-17R domain, a unique intracellular signaling domain found within all IL-17Rs (also named as SEF proteins) as well as the Act1^[81]^, which is essential in IL-17A mediated cell death^[82]^. However, *in vitro* studies of OPCs have found that extracellular-signal-regulated kinase (ERK) protein expression and phosphorylation significantly elevated following IL-17A treatment. This suggests that IL-17A promotes OPCs to differentiate into OLs by activating the ERK1/2 pathway^[29]^. Collectively, even though some of the upregulation (or downregulation) of receptors or other molecules in OL-lineage cells and their related effects have been studied, further exploration is required to understand the underlying mechanisms involved in immunologic changes in OL-lineage cells.

## Scope for immunotherapeutic targeting of OL-lineage cells

Since OL-lineage cells are involved in CNS immune response, we wonder whether immunotherapies could target OL-lineage cells. Sphingosine 1-phosphate receptor (S1PR) modulator Fingolimod can be phosphorylated into FTY720P, an effective element according to several *in vivo* studies^[83–84]^. FTY720P have been found to effectively regulate the immune condition of OPCs in their ability to reciprocally modulate mRNA levels of S1PR1 and S1PR5 in OPCs. This is since short-term treatment as well as long-term treatment show opposite effects^[85]^. This finding may suggest a plausible process where FTY720 initially (and preferentially) binds to S1P5, thereby inducing down-regulation of S1P5, whereby S1P1 is upregulated as a compensatory mechanism. This upregulation allows FTY720 to bind and downregulate S1P1 while upregulating S1P5. This leads to an inhibitory effect on OPCs maturation and rescues OPCs from cellular death.

Another important pathway and potential therapeutic target would be the sonic hedgehog (Shh) pathway. The Shh is of particular interest to researchers because of its role in OPC maturation^[86]^. A study, using a Western blotting technique, found that expression levels of Shh and its receptor smoothened as well as the effector glioma-associated oncogene homolog 1 significantly increased following FTY720P treatment in the CNS of EAE mice. This suggests that this pathway not only promotes OPCs proliferation, but also promotes cellular differentiation. Interestingly, antigen-specific immunotherapeutics which use myelin oligodendrocyte glycoprotein peptides have provided new insights into developing treatments for MS. Myelin oligodendrocyte glycoprotein (MOG) is a myelin antigen which can trigger T cells as well as B cells responses^[87]^. Therefore MOG is an important target for the autoimmune responses because this results in inflammation and demyelination of the CNS.

**Figure 3:**
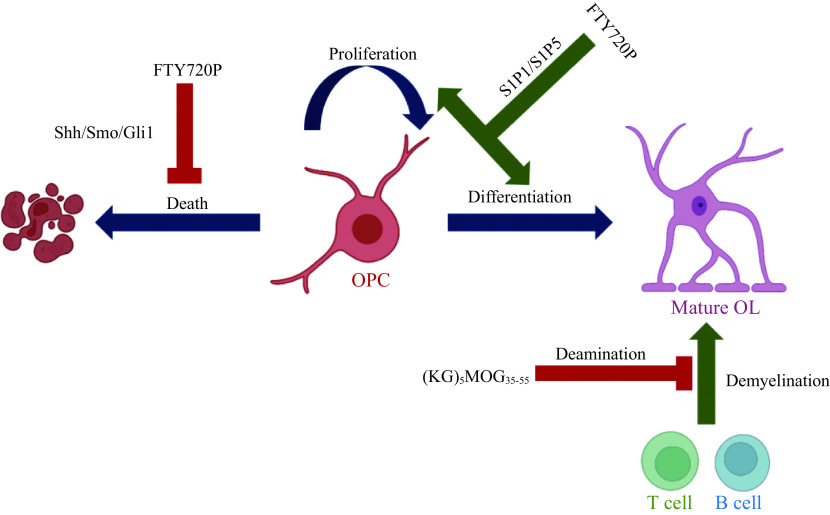
Schematic of OL-lineage cells as immunotherapeutic targets.

Recently, studies have demonstrated that synthesized MOG peptides conjugated with mannan polysaccharides, namely (KG)_5_MOG_35-55_, is a potent approach for MS immunotherapies^[88]^. These peptides form deaminated products in basic conditions, where Asn53 is primarily modified to Asp. In this study, the researchers found that wild type and deaminated derivative peptides conjugated with mannan independently and inhibited the development of neurological symptoms and inflammatory demyelinating spinal cord lesions in MOG_35-55_-induced EAE^[88–90]^. Although, current studies which have identified OL-lineage cells as an immunotherapeutic target are still in the early stages, there is optimism that these maybe used as a treatment for demyelinating diseases in the future (***Fig. 3***).

### Conclusion

OL-lineage cells have long been regarded as simple myelin producers. However, new insights into this cell lineage have shown that its immunologic role is an important aspect in the CNS physiology. In early studies, OPCs and OLs have been identified as targets of immunologic attacks. While studying the role of OL-lineage cells as a "victim" of the immune response, researchers have identified that OL-lineage cells may in fact play a more active immunomodulator role, and therefore can also be "agent". Studies have shown that OPCs and OLs are able to change their immune states by secreting cytokines, chemokines and other neuroinflammatory factors in response to immune attacks. This consequently effects a number of different mechanisms in our immune cells. Collectively, these findings demonstrate the immunologic role of OPCs and OLs, and suggests that for brain pathogens such as MS, OL-lineage cells can be modulated directly by the immune system. At the same time, these cells can in modulate the immune system by secreting different immunologic factors and are therefore also "agent". These insights provide us with a new perspective and suggest we ought to develop targeted immunotherapeutics specifically for demyelinating diseases such as MS.
